# Assessment of Three-Phasic CT Scan Findings of Cirrhosis Due to Primary Sclerosing Cholangitis Versus Cryptogenic Cirrhosis

**DOI:** 10.7759/cureus.23956

**Published:** 2022-04-08

**Authors:** Nazanin Sadraei, Hamed Jafari, Amin Sadraee, Banafsheh Zeinali-Rafsanjani, Hemmatollah Rastgooyan, Aryan Zahergivar

**Affiliations:** 1 Department of Radiology, Medical Imaging Research Center, Shiraz University of Medical Sciences, Shiraz, IRN; 2 Department of Urology, Nephro-Urology Research Center, Shiraz University of Medical Sciences, Shiraz, IRN; 3 Department of Nuclear Medicine and Molecular Imaging, Shiraz University of Medical Sciences, Shiraz, IRN; 4 Department of Radiology, Shiraz University of Medical Sciences, Shiraz, IRN

**Keywords:** abdominopelvic ct scan, cholangiocarcinoma, inflammatory bowel disease, cryptogenic cirrhosis, primary sclerosing cholangitis

## Abstract

Background: The CT findings of cirrhosis caused by primary sclerosing cholangitis (PSC) differ from cryptogenic cirrhosis. PSC could become complicated with biliary cirrhosis and cholangiocarcinoma. This study aimed at augmenting the information on the role of the three-phasic-abdominopelvic CT scan in PSC.

Material and methods: A total of 185 CT scans were retrospectively reviewed, including 100 patients with cryptogenic cirrhosis and 85 patients with PSC-cirrhosis. Different morphologic criteria were compared, including segmental atrophy/hypertrophy, hepatic contour, portal-hypertension, perihilar lymphadenopathy, biliary tree dilatation, gallbladder appearance. Inflammatory-bowel-disease (IBD) and cholangiocarcinoma frequency, presence of perihilar lymph nodes (LNs), and their size during end-stage PSC cirrhosis are investigated.

Results: Six findings occur more frequently with PSC than those diagnosed with cryptogenic cirrhosis. Modified caudate/right lobe (m-CRL) ratio >0.73, moderate and severe lobulated liver contour, lateral left lobe atrophy, over distended gallbladder (GB), biliary tree dilatation and wall thickening, and LN sizes were higher in PSC patients as compared to cryptogenic cirrhosis (P < 0.005). Ascites and portosystemic collateral formations were significant in cryptogenic cirrhosis compared to PSC patients (P < 0.005). Cholangiocarcinoma frequency in PSC patients was 14.7%, and the frequency of inflammatory bowel disease (IBD) was 57.6%. Further, 22.4% of the patients were diagnosed with IBD and PSC simultaneously. The LN number and size in PSC patients were not different between those with or without cholangiocarcinoma.

Conclusion: Using three-phasic CT scans and PSC characteristics could be considered as an additional suggestion besides pathology measures. Diagnosis of PSC based on histological findings could be a last resort due to its invasive essence and specific characteristics of PSC in imaging.

## Introduction

Primary sclerosing cholangitis (PSC) presents in a heterogeneous manner and is characterized by chronic inflammation of the intra/extra bile duct epithelium and multifocal fibrotic biliary duct strictures [1]. It predominantly affects young and middle-aged men starting at 30-40 years of age [2]. PSC prevalence is 0-16.2 per 100,000 inhabitants in any defined geographical area, increasing over time [3]. There is a high correlation between PSC and inflammatory bowel disease (IBD), where 70-80% of patients with PSC were detected to have developed IBD during their lifetime. The age range of 10-15 years is when PSC typically progresses. It eventually leads to biliary cirrhosis and possibly death. The cause of death is presumed to be decompensation of the liver in patients [4]. The PSC complications include hepatic osteodystrophy, development of dominant bile duct strictures, recurrent cholangitis (10-15%), portal hypertension, cirrhosis, and disease-associated malignancies, including cholangiocarcinoma (CCC) [5].

The pathogenesis of PSC is not elucidated; however, many specialists agree on an autoimmune component. Histologic features of PSC are often nonspecific and prone to sampling variations due to the heterogeneous involvement of the biliary tree [5]. A typical but rare histopathologic feature of PSC is substantial periductal ("onion-skin") fibrosis with minimal inflammatory cells [6]. Hence, a biopsy procedure is not recommended for diagnosing typical cholangiographic findings.

PSC diagnosis is usually based on clinical, biochemical, and cholangiography findings [7]. The diagnostic imaging tools for PSC include MR cholangiography, magnetic resonance cholangiopancreatography (MRCP), and endoscopic retrograde cholangiopancreatography (ERCP). The main characteristic findings in MRCP are biliary irregularities with multifocal short segment strictures and dilatations in intra and/or extra bile ducts, which alternate with normal ducts causing "beaded appearance." Peripheral bile ducts are obliterated with advanced fibrosis known as "pruned tree." Other findings include periportal lymphadenopathy, hypertrophy of the caudate lobe, diverticular outpouching, mural irregularity, and isolated involvement of extra-hepatic bile ducts [6-8].

The CT findings in PSC patients consist of contour abnormalities and atrophy, marked caudate lobe hypertrophy, a lower density of the atrophied liver relative to the hypertrophied caudate lobe, and dilated intrahepatic bile duct segments. Other findings include atrophy of the left lateral segments (segments 2 and 3) [8], enlarged perihilar lymph nodes, and increased volume of gallbladder (GB) [9], an abnormally long (greater than 15 mm) common channel, and pancreatic duct abnormalities [8]. Loss of peribiliary vessels is expected in PSC, which can contribute to ischemia, causing slight bile duct loss but is probably secondary to the inflammatory process [10].

Cryptogenic cirrhosis is a term used only after extensive exclusion of substantial etiologies. The prevalence of cryptogenic cirrhosis ranges from 5% to 30% of cirrhotic patients [11]. Several etiologies interpret the underlying causes, such as occult alcohol abuse, non-B non-C viral hepatitis, and non-alcoholic steatohepatitis (NASH) [12]. In Iran, cryptogenic cirrhosis is reported as the most prevalent etiology of cirrhosis, with 59.6% of all the age groups followed by hepatitis B virus [13].

Although previous studies have reported on CT scan findings, there are still many questionable image findings of PSC evaluation. This study aimed to provide more information by addressing the role of three-phasic abdominopelvic CT scan in PSC diagnosis versus cryptogenic cirrhosis. In order to differentiate between PSC-induced cirrhosis and cryptogenic cirrhosis, a spectrum of CT scan findings was analyzed in 85 patients with end-stage cirrhosis caused by PSC and 100 cryptogenic cirrhotic patients.

## Materials and methods

Surveillance program

The university ethics committee approved this retrospective study (reference number: 15942). Shiraz Namazi Hospital is a tertiary center for diagnosis, treatment, and liver transplantation, in which cirrhotic patients with various causes are admitted. Patients with cryptogenic cirrhosis and previously histopathologic proven PSC were selected from 2011 to 2017.

Patient selection

Overall, 100 patients with cryptogenic cirrhosis consisting 59 males and 41 females (mean age = 50.2 years: range = 15-74 years), and 85 individuals with clinical and pathological diagnosis of PSC consisting 59 males and 26 females (mean age = 40.8; range = 18-68) were studied. All patients were candidates for liver transplantation.

Patients with biopsy-proven histological diagnosis of PSC-cirrhosis, who had no viral, autoimmune, and hereditary disease of the liver and were identified as cryptogenic cirrhosis, and for whom three-phasic abdominopelvic CT scan was available at the hospital Picture Archiving and Communication System (PACS) were included. Patients with no definite diagnosis of PSC, who were positive for viral, autoimmune, or hereditary causes of cirrhosis, and without three-phasic abdominopelvic CT scan were excluded from this study.

Of 100 patients with biopsy-proven histological diagnosis of PSC, 85 met our inclusion criteria. Cryptogenic cirrhotic patients included in this study consisted of 100 cases from 150 in the surveillance data center. The rest of the patients were excluded from the study due to insufficient available data on the patients’ profiles. 

Imaging techniques

Three-phasic dynamic CT scans were obtained by Bright Speed 16 (Milwaukee, WI: GE Medical Systems). The CT scans used in this study were in the arterial phase (about 30 seconds), equilibrium (about 60 seconds), and delayed phase (five to seven minutes). The scans were performed using 120 kVp with a slice thickness of 1.25-2.0 mm.

Visipaque 320 mg and Omnipaque 300 mg, as two nonionic contrast agents, were injected using two dual CT injectors (Medrad {Saxonburg, PA: Bayer Radiology} and Optivantage {Saint Louis, MO: Liebel-Flarsheim Company LLC}) at a dose of 1.5-5 ml/kg (maximum: 100 ml) with the flow rate of 2.5-3 ml/sec through the peripheral antecubital vein. Two radiology specialists with 15 years of experience reviewed the CT scan images. A third reader resolved discordant, difference of opinion. The frequency of several specific findings in patients with cirrhosis caused by PSC versus findings in cryptogenic cirrhosis was analyzed.

Data collection

**Figure 1:**
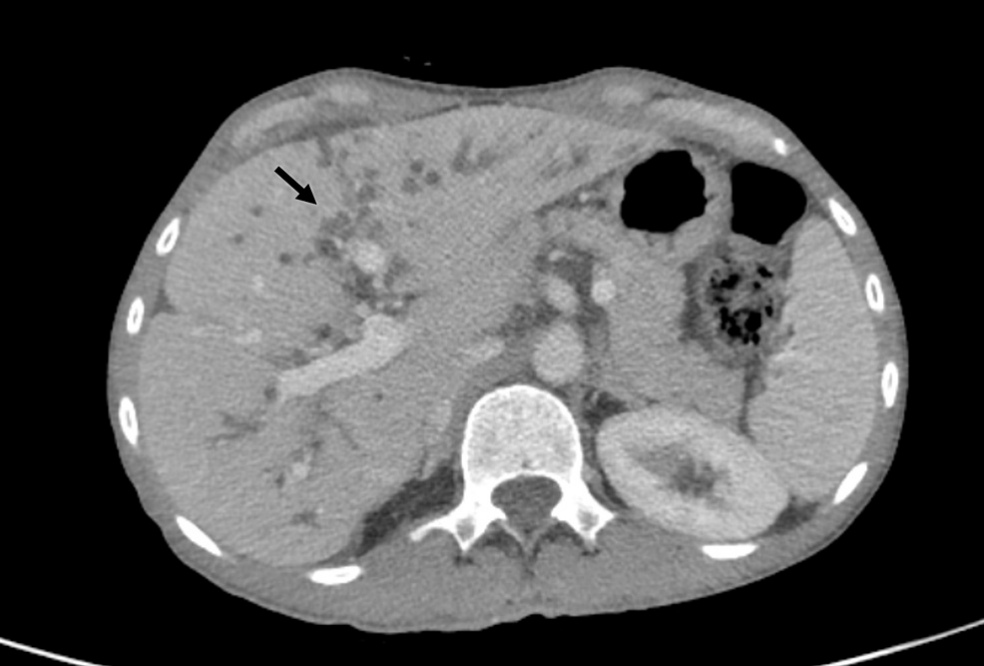
Three-phasic CT scan in the portovenous phase of a 34-year-old female, which is a known case of PSC, referred for liver transplantation. The beaded appearance of biliary tree is noted. PSC: primary sclerosing cholangitis

**Figure 2:**
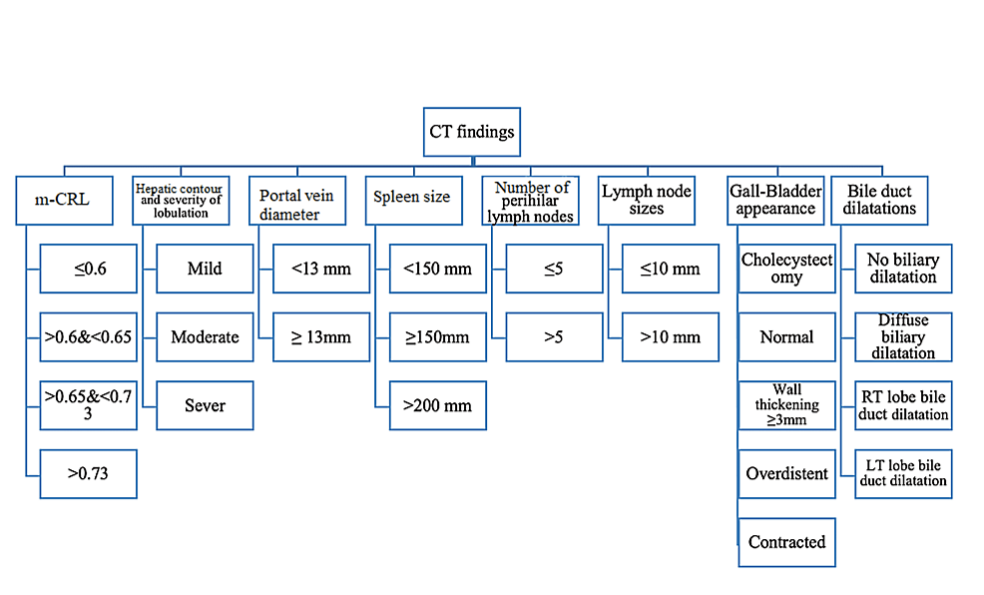
Some CT scan findings which were considered in this study and their subcategories. m-CRL: modified caudate/right lobe; RT: right

The findings included evaluation of segmental atrophy and hypertrophy, hepatic contour, assessment of associated portal hypertension (by portal vein size, spleen size, ascites, and presence of portosystemic collateral pathways), presence and size of perihilar lymphadenopathy, radiologic findings of intrahepatic bile duct dilatation (>3 mm in anteroposterior {AP} diameter), bile duct thickening and gallbladder appearance, biopsy-proven cholangiocarcinoma, and IBD. Some of the CT findings and their subcategories are shown in Figures 1, 2.

**Figure 3:**
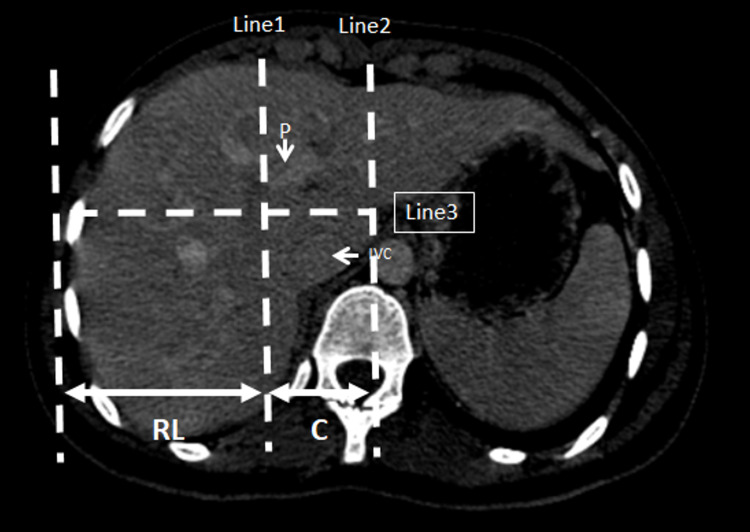
CT scan showing modified caudate-right lobe ratio. Line 1 passes through the right lateral wall of the right portal vein bifurcation and parallel to the midsagittal body plane. Line 2 passes through the caudate lobe’s most medial margin and is parallel to line 1. Line 3 is drawn perpendicular.

Segmental atrophy and hypertrophy, measured by modified caudate/right lobe {m-CRL} ratio, were subdivided into four categories (Figures 2, 3) [14]. The hepatic contour with one nodule greater than 3 cm diameter was assumed lobular, and the severity of lobulation was categorized subjectively to mild, moderate, or severe [8]. Portal vein diameter was measured as the anteroposterior (AP) diameter in the axial view. The portal vein diameter equal to or greater than 13 mm was considered portal hypertension, otherwise normal.

Splenic size of 150 mm or greater, measured from the coronal view, was considered splenomegaly, whereas splenic size larger than 200 mm was recorded as massive splenomegaly. The presence/absence of ascites and portosystemic collateral pathways was considered an indicator of portal hypertension. The perihilar lymph node counts were divided into less than or equal to five and more than five. Lymph node sizes were measured in short axis diameter perspective and categorized into two groups of more than 10 mm and less than or equal to 10 mm (Figure 2).

Gallbladder appearance, classified into five categories, was assessed in nothing by mouth (NPO) patients for six hours (Figure 2). Bile duct dilatations were classified into four groups including, no biliary dilatation, diffuse biliary dilatation, right (RT) lobe bile duct dilatation, and left (LT) lobe bile duct dilatation predominance (Figure 2).

Statistics

The data were subjected to statistical analysis using the Chi-squared goodness of fit and t-test statistics (two-tailed) where appropriate. All the data were put into IBM SPSS Statistics for Windows version 15.0 (Armonk NY: IBM Corp). The level of significance was set at P ≤ 0.05. We used descriptive analysis to describe the data, including mean ± standard deviation (SD) for quantitative variables and frequency (percentage) for categorical variables.

## Results

**Figure 4:**
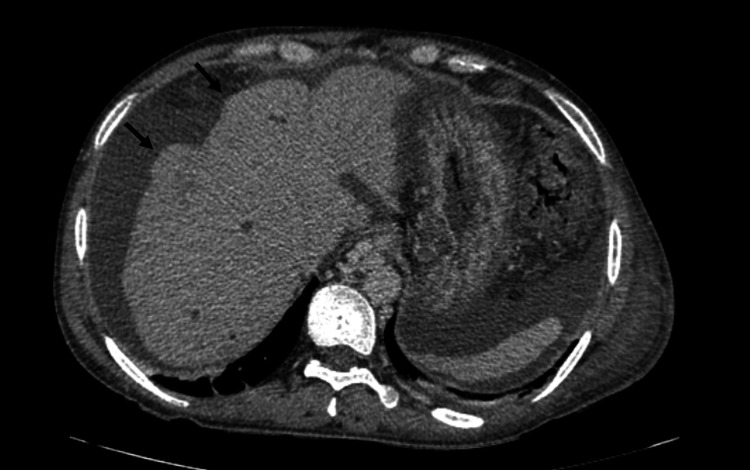
Three-phasic CT scan in portovenous phase of a 57-year-old male, which is a known case of PSC, referred for liver transplantation. The lobulated border is observable. PSC: primary sclerosing cholangitis

The frequency distribution of the findings in patients with primary sclerosing cholangitis versus cryptogenic cirrhosis is presented in Table 1. Six findings were more frequent in patients with PSC compared with cryptogenic cirrhosis. The m-CRL ratio in PSC patients was greater than 0.73. Moderate and severe lobulated liver contours were higher in PSC patients (P < 0.001) (Figure 4). The lateral left lobe atrophy, including segments 2 and 3, was also more frequent in PSC patients (P < 0.001). Cholecystectomy, normal-appearing GB, and over-distended GB were more frequent in PSC patients than cryptogenic cirrhosis; however, wall thickening and contracted GB were higher in cryptogenic cirrhotic patients (P < 0.001). Biliary tree dilatation and wall thickening were also more frequent in patients with PSC (Table 1). The frequencies of the lymph node size more than 10 mm were higher in PSC patients than in cryptogenic cirrhosis (P < 0.005). Portal vein size and splenomegaly were not different between the two groups of patients. Ascites and collateral formations were more prevalent in cryptogenic cirrhosis as compared to PSC (P < 0.005).

**Table 1:** Frequency of CT findings and their subcategories among PSC and cryptogenic cirrhosis patients. m-CRL: modified caudate/right lobe; PSC: primary sclerosing cholangitis

CT findings	Subcategories	PSC patients N=84 (%)	Cryptogenic cirrhosis N=100 (%)	P-value
m-CRL ratio	≤0.6	31 (36.5%)	36 (36%)	<0.001
	>0.6, ≤0.65	7 (8.2%)	37 (37%)	
	>0.65, ≤0.73	11 (12.9%)	12 (52.2%)	
	>0.73	36 (42.4%)	15 (29.4%)	
Gallbladder appearance	Cholecystectomy	23 (27.1%)	1 (1%)	<0.001
	Normal	28 (32.9%)	12 (12%)	
	Wall thickening	18 (21.2%)	30 (30%)	
	Overdistent	16 (18.8%)	9 (9%)	
	Contracted	0 (0%)	48 (48%)	
BD dilatation	Both lobes	51 (60%)	7 (7%)	<0.001
	Left side	23 (27.1%)	0 (0%)	
	Rt side	3 (3.5%)	0 (0%)	
	No	8 (9.4%)	93 (93%)	
Lymph node size	≤10mm	13 (15.3%)	33 (33%)	0.005
	>10mm	72 (84.7%)	67 (67%)	
Hepatic contour and severity of lobulation	Mild	27 (31.8%)	55 (57.3%)	0.002
	Moderate	48 (56.5%)	36 (37.5%)	
	Severe	10 (11.8%)	5 (5.2%)	
LT lobe	Normal	36 (42.4%)	39 (39%)	<0.001
	Lateral lobe Atrophy	49 (57.6%)	20 (20%)	
	Total atrophy	0 (0%)	41 (41%)	
Portal vein diameter	<13mm	26 (30.6%)	13 (13%)	>0.005
	≥13mm	57 (67.1%)	81 (81%)	
	Thrombosed	2 (2.4%)	6 (6%)	
Spleen size	<150mm	44 (51.8%)	32 (32%)	<0.005
	≥150mm	29 (34.1%)	42 (42%)	
	≥200mm	11 (12.9%)	22 (22%)	
	Splenectomy	1 (1.2%)	4 (4%)	
Ascites	Yes	31 (36.5%)	63 (63%)	0.001
	No	54 (63.5%)	37 (37%)	
Collaterals	Yes	28 (32.9%)	63 (63%)	<0.001
	No	57 (67.1%)	37 (37%)	

**Figure 5:**
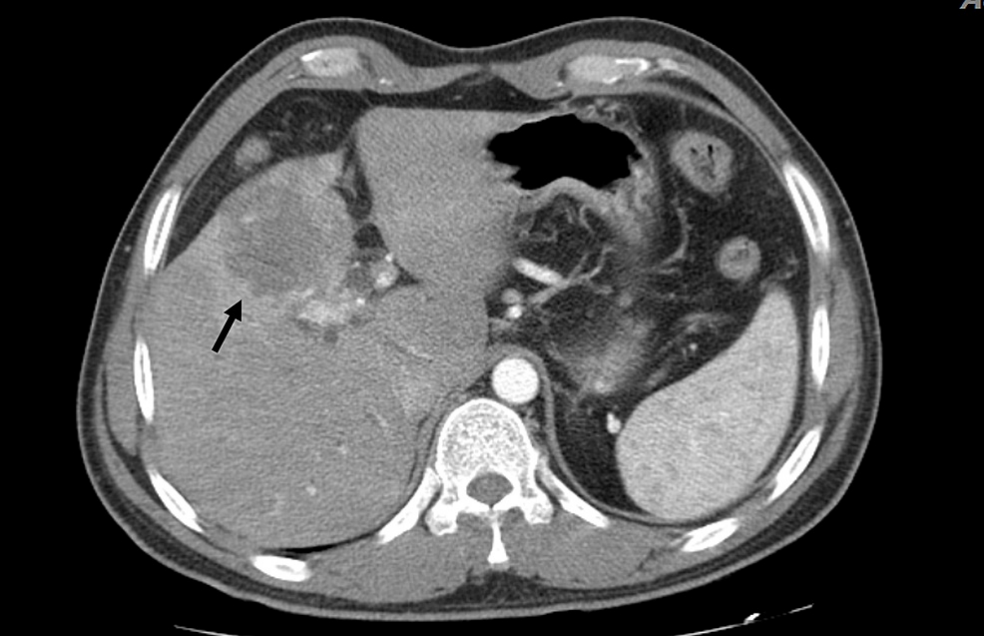
Three-phasic CT scan in the arterial phase of a 45-year-old male, who was a known case of PSC, presented with jaundice. The heterogeneous mass lesion in the medial segment of LT lobe of the liver was associated with capsule retraction and delayed enhancement, indicating cholangiocarcinoma. PSC: primary sclerosing cholangitis; LT: left

As shown by pathological observations, cholangiocarcinoma was detected in 12 cases of PSC-cirrhosis (14.7%). About 57.6% of PSC patients had a history of long-term IBD, and 22.4% of the patients were diagnosed with IBD and PSC simultaneously (Figure 5). The lateral left lobe atrophy was more frequently detected (P < 0.005) in PSC patients with an m-CRL ratio greater than 0.73. The number and size of lymph nodes (LNs) in PSC patients were not different from those with or without cholangiocarcinoma.

## Discussion

PSC, a rare liver disease with unknown etiology, is associated with IBD and can result in catastrophic conditions such as cirrhosis and cholangiocarcinoma [10]. Cryptogenic cirrhosis is the leading cause of cirrhosis in Iran, making the diagnosis of PSC based on a CT scan and differentiating it from other causes more crucial.

Up to 1999, cross-sectional findings of PSC-induced cirrhosis were described as nodularity of hepatic margin, atrophy of RT lobe associated with hypertrophy of LT lobe. Albeit considerable overlap in image findings of cirrhosis caused by PSC versus other factors is seen, some have unique features such as Budd-Chiari syndrome [15]. However, in an article published by Dodd et al. in 1999, different hepatic morphologies in PSC-induced cirrhosis compared to other factors causing cirrhosis were suggested [8]. These features included severity of contour lobularity, lateral segment atrophy, post segment atrophy, caudate lobe hypertrophy, bile duct dilatation, and biliary calculi.

Out of the 85 cases of PSC patients in this study, 49 cases (57.6%) had associated IBD, while cholangiocarcinoma was diagnosed in only 12 patients (14.7%). In the study by Gulamhusein et al., 399 patients with PSC-IBD were investigated. Colectomy was done for 137 (34%), and 123 patients were found to have had developed CCC (30%) [16]. In our study, 11 patients with positive IBD had cholangiocarcinoma (22.4%), and IBD was not diagnosed in only one case with CCC (2.8%). In the present study, the lobar atrophy and hypertrophy by m-CRL ratio were also investigated and yielded the following unique finding. The m-CRL > 0.73 was associated with 42% of PSC-caused cirrhosis compared with 15% in cryptogenic cirrhosis patients.

According to Ruiz et al., in MRIs of 57.8% of PSC patients, parenchymal dysmorphia was regarded as lobulated contour [17]. In the study of Dodd et al., mild-to-moderate lobulated contour was noted in 20 PSC patients (56%) and 55 (12%) patients diagnosed with cirrhosis due to other causes; severe lobulation was reported in six (17%) PSC patients and five (1%) patients with cirrhosis (P = 0.01). This finding is in line with our findings in which 27 PSC patients (31.8%) and 55 cryptogenic cirrhosis ones (57.3%) depict mild lobular contour. Some 48 PSC patients (56.5%) and 36 (37.5%) cryptogenic cirrhosis patients had moderated lobular contour (P = 0.002). A severe case of lobulated contour was seen in 10 (11.8%) PSC patients and five (5.2%) cryptogenic cirrhosis ones (P = 0.002). This variation from the previous study could have resulted from PSC duration that caused cirrhosis.

Caldwell et al., reporting focal atrophy of segments 2 and 3 in 4 out of 44 patients, concluded that this finding is sometimes early and subtle in PSC [18]. It was also demonstrated that mild to moderate lateral lobe atrophy was the issue in 6 PSC patients (17%) and four cirrhotic patients of other causes (1%). Furthermore, severe lateral segment atrophy in 4 (11%) PSC patients and 10 (2%) cirrhotic patients was reported [8]. The present study was congruent with the studies mentioned above. Left lobe atrophy was present in 49 PSC patients (57.6%) in comparison to 20 patients (20%) with cryptogenic cirrhosis (P < 0.001). Left lateral lobe atrophy could be due to bile duct anatomy and predilection of involvement and obstruction in the left lateral lobe. Furthermore, cryptogenic cirrhosis diffused bile ducts involvement was observed [19].

Van de Meeberg et al. reported significant enlargement of gallbladder volume in PSC patients [9]. In our study, over-distended GB was seen in 16 PSC patients (18.8%), whereas only nine patients (9%) with cryptogenic cirrhosis demonstrated this feature. No contracted GB was found in PSC patients, although this occurred in 48 patients (48%) with cryptogenic cirrhosis (P < 0.001).

In cryptogenic cirrhosis pathogenesis, the destruction of hepatocytes results in decreased bile production. However, in PSC, obstruction and inflammation of the biliary tree occur before the hepatocyte's destruction. Bile production could last further until the end stages; thus, GB contraction would be seen much less in PSC cases [20]. Biliary tree dilatation in the study of Ruiz et al. was found in 50 (78.1%) of the last MRIs of PSC patients [17]. In a study by Dodd et al., similar findings were obtained in 24 patients (67%) in comparison to 26 (6%) patients with other causes of cirrhosis [8].

Outwater et al. illustrated lymph node enlargement in 13 out of 20 PSC patients (65%) [21]. A similar outcome was obtained in the current study. LN size of more than 10 mm was found in 72 PSC patients (84.7%) and 67 cryptogenic cirrhosis (67%). LN size of less than10 mm was recorded in 13 PSC patients (15.3%) vs. 33 cryptogenic cirrhosis (33%). 

End-stage cirrhosis of any cause can lead to portal vein hypertension due to parenchymal fibrosis, and it can be measured by portal vein size, splenomegaly, and presence of ascites and portosystemic collateral pathways. As shown in Table 2, ascites and portosystemic collateral pathway formations were significantly higher in cryptogenic cirrhosis than in PSC patients. No significant relationship was noted in portal vein size and splenomegaly between these two groups. Portosystemic collateral pathway formation and ascites were much more common in cryptogenic cirrhosis patients (P < 0.001). The underlying cause could be that PSC-induced biliary tree obstruction appears sooner than cryptogenic cirrhosis. Due to the chronic nature of cryptogenic cirrhosis, the manifestation of portosystemic collateral pathway formation is probable [22].

**Table 2:** PSC with CCC vs PSC without CCC comparison in size and number of lymph nodes. CCC: cholangiocarcinoma; PSC: primary sclerosing cholangitis; LN: lymph node

PSC patients	Cholangiocarcinoma		P-value
	No	Yes	
LN≤5mm	25 (29.4%)	3 (3.5%)	0.005
LN>5mm	48 (56.5%)	9 (10.6%)	
LN≤10mm	11 (12.9%)	2 (2.4%)	0.005
LN >10mm	62 (72.9%)	10 (11.8%)	

Lymph node presence and size were evaluated in PSC patients with CCC compared to those in PSC patients without CCC (Table 2). Kirchner et al. showed that enlarged perihilar lymph nodes were presented in 73% (60/82) of PSC patients without CCC versus 86% (30/35) of patients with CCC; however, the difference was not significant [23]. In the current study, more than five perihilar LNs were seen in 48 PSC patients (56.5%) without CCC and in nine PSC patients with CCC (10.6%). Perihilar LNs equal and less than five were observed in 25 (29.4%) PSC patients without CCC and three (3.5%) patients with CCC (P = 0.528). 

Due to biliary tree involvement in PSC cases, inflammation and cholangitis could be superimposed, resulting in increasing the lymph node size, thus making no difference in lymphadenopathy with or without CCC [24]. In the previous study, which was based on ultrasound evaluation of the PSC patients with CCC, the width of lymph nodes was significantly larger than patients without CCC (12±6 mm versus 8±4 mm; P < 0.001). However, it was concluded that perihilar lymph nodes were not suggestive of CCC, which agrees with our study [23]. Perihilar LN size was less than or equal to 10 mm in 11 patients with no CCC PSC (12.9%) and two PSC patients with CCC (2.4%), whereas LNs larger than 10 mm was found in 62 CCC PSC patients (72.9%) and 10 PSC patients with CCC (11.8%). Lymphadenopathy can be seen in PSC patients due to causes other than cholangiocarcinoma; therefore, lymph node size and numbers do not vary significantly in PSC with cholangiocarcinoma as opposed to PSC without cholangiocarcinoma [25].

Primary sclerosing cholangitis (PSC) might lead to cirrhosis, leading to more severe conditions like cholangiocarcinoma. Pathologic findings cannot verify PSC diagnosis efficiently; therefore, utilizing radiological findings is suggested. In this study, more specific findings were obtained for PSC-caused cirrhosis versus cryptogenic cirrhosis, such as the left lobe predominance and diffused biliary dilatation, moderate contour lobulation, over-distended gallbladder, and left lateral lobe atrophy in addition to the m-CRL ratio greater than 0.73. Further studies are suggested on the anatomical variation of the biliary tree, portal vein, and hepatic artery in cirrhosis caused by PSC versus other causes.

## Conclusions

Primary sclerosing cholangitis (PSC) might lead to cirrhosis, leading to more severe conditions such as cholangiocarcinoma. Histological findings cannot verify PSC diagnosis efficiently; therefore, utilizing radiological findings prior to any invasive procedure is strongly encouraged. In this study, more specific results were obtained for PSC-caused cirrhosis versus cryptogenic cirrhosis, such as the left lobe predominance and diffused biliary dilatation, moderate contour lobulation, over-distended gall bladder, and left lateral lobe atrophy in addition to m-CRL ratio greater than 0.73. Further studies are suggested on the anatomical variation of the biliary tree, portal vein, and hepatic artery in cirrhosis caused by PSC versus other causes.

## References

[1] ElNayal A, Tsatoumas M, Alsubhi M, Saif S, Alaref A, Reinhold C (2016). Review of radiologic manifestations in primary and secondary sclerosing cholangitis. https://epos.myesr.org/poster/esr/ecr2016/C-1346.

[2] Abdalian R, Dhar P, Jhaveri K, Haider M, Guindi M, Heathcote EJ (2008). Prevalence of sclerosing cholangitis in adults with autoimmune hepatitis: evaluating the role of routine magnetic resonance imaging. Hepatology.

[3] Boonstra K, Beuers U, Ponsioen CY (2012). Epidemiology of primary sclerosing cholangitis and primary biliary cirrhosis: a systematic review. J Hepatol.

[4] Eksteen B (2016). The gut-liver axis in primary sclerosing cholangitis. Clin Liver Dis.

[5] Eaton JE, Talwalkar JA, Lazaridis KN, Gores GJ, Lindor KD (2013). Pathogenesis of primary sclerosing cholangitis and advances in diagnosis and management. Gastroenterology.

[6] Seo N, Kim SY, Lee SS, Byun JH, Kim JH, Kim HJ, Lee MG (2016). Sclerosing cholangitis: clinicopathologic features, imaging spectrum, and systemic approach to differential diagnosis. Korean J Radiol.

[7] Gotthardt D, Chahoud F, Sauer P (2011). Primary sclerosing cholangitis: diagnostic and therapeutic problems. Dig Dis.

[8] Dodd GD 3rd, Baron RL, Oliver JH 3rd, Federle MP (1999). End-stage primary sclerosing cholangitis: CT findings of hepatic morphology in 36 patients. Radiology.

[9] van de Meeberg PC, Portincasa P, Wolfhagen FH, van Erpecum KJ, VanBerge-Henegouwen GP (1996). Increased gall bladder volume in primary sclerosing cholangitis. Gut.

[10] Tischendorf JJ, Hecker H, Krüger M, Manns MP, Meier PN (2007). Characterization, outcome, and prognosis in 273 patients with primary sclerosing cholangitis: a single center study. Am J Gastroenterol.

[11] Kodali VP, Gordon SC, Silverman AL, McCray DG (1994). Cryptogenic liver disease in the United States: further evidence for non-A, non-B, and non-C hepatitis. Am J Gastroenterol.

[12] Caldwell SH, Oelsner DH, Iezzoni JC, Hespenheide EE, Battle EH, Driscoll CJ (1999). Cryptogenic cirrhosis: clinical characterization and risk factors for underlying disease. Hepatology.

[13] Hatami B, Ashtari S, Sharifian A, Rahmani Seraji H, Khalili E, Hatami Y, Zali MR (2017). Changing the cause of liver cirrhosis from hepatitis B virus to fatty liver in Iranian patients. Gastroenterol Hepatol Bed Bench.

[14] Furusato Hunt OM, Lubner MG, Ziemlewicz TJ, Muñoz Del Rio A, Pickhardt PJ (2016). The liver segmental volume ratio for noninvasive detection of cirrhosis: comparison with established linear and volumetric measures. J Comput Assist Tomogr.

[15] Awaya H, Mitchell DG, Kamishima T, Holland G, Ito K, Matsumoto T (2002). Cirrhosis: modified caudate-right lobe ratio. Radiology.

[16] Gulamhusein AF, Eaton JE, Tabibian JH, Atkinson EJ, Juran BD, Lazaridis KN (2016). Duration of inflammatory bowel disease is associated with increased risk of cholangiocarcinoma in patients with primary sclerosing cholangitis and IBD. Am J Gastroenterol.

[17] Ruiz A, Lemoinne S, Carrat F, Corpechot C, Chazouillères O, Arrivé L (2014). Radiologic course of primary sclerosing cholangitis: assessment by three-dimensional magnetic resonance cholangiography and predictive features of progression. Hepatology.

[18] Caldwell SH, Hespenheide EE, Harris D, de Lange EE (2001). Imaging and clinical characteristics of focal atrophy of segments 2 and 3 in primary sclerosing cholangitis. J Gastroenterol Hepatol.

[19] Al-Sukhni W, Gallinger S, Pratzer A (2008). Recurrent pyogenic cholangitis with hepatolithiasis--the role of surgical therapy in North America. J Gastrointest Surg.

[20] Popper H (1986). General pathology of the liver: light microscopic aspects serving diagnosis and interpretation. Semin Liver Dis.

[21] Outwater E, Kaplan MM, Bankoff MS (1992). Lymphadenopathy in sclerosing cholangitis: pitfall in the diagnosis of malignant biliary obstruction. Gastrointest Radiol.

[22] Tsochatzis EA, Bosch J, Burroughs AK (2014). Liver cirrhosis. Lancet.

[23] Kirchner GI, Tischendorf JJ, Bleck J (2008). Perihilar lymph nodes in patients with primary sclerosing cholangitis with and without cholangiocellular carcinoma. Scand J Gastroenterol.

[24] Johnson KJ, Olliff JF, Olliff SP (1998). The presence and significance of lymphadenopathy detected by CT in primary sclerosing cholangitis. Br J Radiol.

[25] Khan SA, Davidson BR, Goldin R (2002). Guidelines for the diagnosis and treatment of cholangiocarcinoma: consensus document. Gut.

